# SARS-CoV-2 at the human-animal interphase: A review

**DOI:** 10.1016/j.heliyon.2021.e08496

**Published:** 2021-11-27

**Authors:** Elmoubasher A. Farag, Md Mazharul Islam, Khalid Enan, Abdel-Rahim M. El-Hussein, Devendra Bansal, Mohamed Haroun

**Affiliations:** a Ministry of Public Health, Doha, Qatar; b Department of Animal Resources, Ministry of Municipality and Environment, Doha, Qatar; c Central Laboratory, Ministry of Higher Education and Scientific Research, Khartoum, Sudan

**Keywords:** COVID-19, SARS-CoV-2, Human-animal interface, Control

## Abstract

Since its emergence in China in December 2019, COVID-19 remains the recent leading disease of concern drawing the public health attention globally. The disease is known of viral origin and zoonotic nature originating from animals. However, to date neither the source of the spillover nor the intermediate hosts are identified. Moreover, the public health situation is intermittently aggravated by identification of new animals susceptible to the SARS-CoV-2 infection, potentially replicating the virus and maintaining intra and interspecies spread of the disease. Although the role of a given animal and/or its produce is important to map the disease pattern, continuous efforts should be undertaken to further understand the epidemiology of SARS-CoV-2, a vital step to establish effective disease prevention and control strategy. This manuscript attempted to review updates regarding SARS-CoV-2 infection at the human-animal interface with consideration to postulations on the genetic relatedness and origin of the different SARS-CoV-2 variants isolated from different animal species. Also, the review addresses the possible role of different animal species and their produce in transmission of the disease. Also, the manuscript discussed the contamination potentiality of the virus and its environmental stability. Finally, we reviewed the currently instituted measures to prevent and manage the spread of SARS-CoV-2 infection. The manuscript suggested the One Health based control measures that could prove of value for the near future.

## Highlights

• Eleven animal species over 30 countries naturally contract SARS-CoV-2 infection.
• Minks establish anthropozoonosis to SARS-COV-2 infection.
• A lot is behind to reveal about SARS-CoV-2 at the human-animal interface.

## 1 Introduction

Emerging infectious diseases are serious global concern due to their impact on world health and economy [1]. Worldwide, over 1.6 million mammalian viruses are estimated to exist in nature, of which 700 are estimated to potentially infect humans [2]. At present, there are more than 60% of known human pathogens, of which 75% are associated with emerging diseases that originate in animals [1, 3].

Over years, human coronavirus (HCoV) has passed through a sequence of expedited evolutionary stages [4] which, although clinically manageable under certain circumstances, it was difficult under other conditions [5]. As such, it is not surprising that coronavirus-2019 disease (COVID-19) has evolved as a global pandemic. Further, because of the high infectious properties of the virus, the disease has been prioritized as public health emergency of international concern [6, 7].

Coronaviruses (CoVs) belong to the family *Coronaviridae* that are divided into four genera: alpha, beta, gamma and delta infecting human and animals [8] Seven members of which are identified as human coronaviruses (HCoVs). All currently known disease producing HCoVs belong to α-CoVs (HCoV-229E and HCoV-NL63) or β-CoVs (HCoV-HKU1, HCoV-OC43, SARS-CoV, MERS-CoV and SARS-CoV-2) [8]. Collectively, HCoV-229E, HCoV-NL63, HCoV-HKU1 and HCoV-OC43 are responsible for approximately one-third of the common infections among humans. However, SARS-CoV, MERS-CoV and SARS-CoV-2 are known to produce asymptomatic, mild or severe respiratory syndromes that can potentially end with 10%, 35% and 6% fatality rates, respectively [9, 10, 11, 12, 13, 14, 15]. SARS-CoV and MERS-CoV are zoonotic in nature and known to infect civet cats and camels, respectively [16, 17]. However, the first reported spillover of SARS-CoV-2 from animals to humans was recorded in Wuhan, China, in 2019 [18]. Since then, the disease spread throughout the world at an unprecedented rate. The affliction of over 215 countries with a case fatality rate of 6% [15] has forced the World Health Organization (WHO) to declare the disease a global pandemic on 11 March 2020 [19]. The International Committee on Taxonomy of Viruses named the disease Coronavirus disease-19 (COVID-19) and the virus as Severe Acute Respiratory Syndrome Coronavirus-2 (SARS-CoV-2) [20].

### 1.1 Clinical remedies for SARS-CoV-2 infections

The International Labor Organization (ILO) [21] and the United Nations (UN) [22] has labeled the ongoing pandemic as the worst global crisis since the Second World War. Currently, there is no single treatment available for COVID-19. However, several clinical trials are underway to assess the efficacy of these drugs to treat COVID-19 or to reduce its clinical consequences. Based on ability to inhibit production of infectious virus particles, several anti-viral replication agents are recommended to treat COVID-19, however, each of which shows various level of efficacy. These anti-viral agents include fusion inhibition agents, protease inhibition agents, transcription inhibition agents, neuraminidase inhibition agents, and M2 ion channel protein blocking agents.

Baricitinib was introduced as a potent SARS-CoV-2 fusion inhibitor retarding the function of AP2-associated protein kinase 1 [23, 24] and thereby prevent viral endocytosis and subsequent viral assembly [24]. Umifenovir (arbidol) is another prescribed fusion inhibiting agent, however, it acts by inhibiting the activity of the hemagglutinin envelope glycoproteins of the virus [25]. Casmostat mesylate, is a third known fusion inhibitor. As a serine protease inhibitor, it has the potential to prevent viral attachment and fusion to the ACE-2 receptors alone or also to the TMPRSS2 receptors [26].

Among SARS-CoV-2 protease inhibitors, lopinavir was the first to be used against COVID-19 as a therapy [27, 28]. However, combing lopinavir-ritonavir was found to be most effective reducing the viral load thereby alleviating the clinical symptoms [25]. Alone or combined with interferon (INF)-β, the lopinavir-ritonavir combination was recommended by WHO as an option for clinical trials against COVID-19 [29]. The triple-drug combination of lopinavir-ritonavir and umifenovir was also observed to substantially reduce lung damage [30]. However, clinically, atazanavir is shown to be more potent anti-viral protease inhibitor in comparison to lopinavir [31]. Similarly, other protease inhibitors such as saquinavir and saquinavir-similar structural agents like indinavir, amprenavir, nelfinavir are also expected to inhibit subsequent viral infection among SARS-CoV-2-infected patients [32, 33] either when used alone or in combination with other anti-SARS-CoV-2 agents [34]. Although darunavir was used in Italy and was reported to be effective against SARS-CoV-2 [35, 36, 37] it was not proved effective when used for patients co-infected with HIV [38].

To date, the most popular and widely known anti-SARS-CoV-2 drug is remdesivir [39, 40, 41], a nucleotide analogue effective against several single stranded RNA viruses at the level of reverse transcription. Under the same umbrella of methodology of inhibition, guanine derivative analogues including favipiravir (avigan) [42, 43, 44] and ribavirin [45] are also used potentially for treating COVID-19. However, different combinations of the previously described anti-viral agents were also clinically tried and recommended to treat COVID-19 [46, 47].

Oseltamivir, a neuraminidase inhibitor previously used for treating influenza infections [48] has also been prescribed as an anti-SARS-CoV-2 agent [49, 50, 51, 52]. Later, a computational study proved the potentiality of oseltamivir for treating SARS-CoV-2 infections [53]. The most recently introduced neuraminidase inhibitors including raltegravir and paritaprevir inhibiting 3CL^Pro^ activity [54] and the medicinal plant phytochemicals inhibiting 3CL^pro^ of the SARS-CoV-2 [55] should also warrants attention.

Potent M2 ion channel protein blockers including amantadine, adamantane, and rimantadine inhibiting virus entry were also prescribed for treating SARS-CoV-2 infections [56, 57]. These drugs work by blocking the protein channel through disruption of the lysosomal gene expression [56]. However, more clinical trials are required to prove their efficacy.

Some previously recognized drugs with no known anti-viral properties have also been shown to possess anti-viral activity and were used against SARS-CoV-2 infections. The famous of which is ivermectin, a potent anti-parasitic agent, has been reported to inhibit importin α/β1-mediated nuclear replication in SARS-CoV-2 [58, 59]. Despite the initial promising use of chloroquine and hydroxychloroquine to treat COVID-19, however, to date, both drugs did not prove to have either prophylactic or curative impact on infection against SARS-CoV-2 [60].

Antibody-based immunotherapeutics have also shown efficacy in both prophylactical and therapeutical protocols against COVID-19. SARS-CoV-2 specific convalescent plasma [61, 62, 63], IFN-α/β [64,65] and IL-6R [34,66] are reported to reduce viral loads and alleviate clinical symptoms among SARS-CoV-2-infected patients. The use of monoclonal antibody preparations including bamlanivimab, casirivimab and imedvimab for SARS-CoV-2-infected children and adolescents was found to be of great value for severe cases requiring hospitalization [67]. However, the use of neutralizing antibodies does not prove efficiency till now.

Several anti-inflammatory agents have also been used alone or in combination with other anti-viral agents to treat SARS-CoV-2 infections. Most famous among these is dexamethasone, which has been prescribed to combat the effect of cytokines storm associated with overproduction of pro-inflammatory cytokines among some COVID-19 patients.

Despite several human vaccines are now on use for primary prevention against COVID-19, it is still uncertain when the currently developed vaccines be available in every part of the world. Moreover, it is still uncertain that these vaccines would be effective against the constantly developing virus new variants. Another major challenge in controlling SARS-CoV-2 and managing its transmission is that, unlike SARS-CoV or MERS-CoV, the virus transfers from human to human before developing the symptoms or clinical signs [68, 69]. This microbial characteristic of SARS-CoV-2 is proving very demanding in local and international control of the virus. For that, the interim measures to hamper the speed of the outbreak remain practicing social distancing, masking and personal hygiene [68, 69].

SARS-CoV-2 is considered a zoonotic in nature and animal(s) serve as its reservoir and intermediate hosts. Currently, intense investigations are being conducted to identify the primary host for the SARS-CoV-2. Presently, there are few studies that investigated the role of animals as the origin of SARS-CoV-2 infection [70, 71]. Several hypotheses have been formulated around the role of animals sold in the Wuhan market in the emergence and spread of SARS-CoV-2 to human. The first of these theories suggests introduction of the virus to the human population from an animal source at the Wuhan market. The second theory suggests that the virus was first introduced to the market from an infected human who transmitted the virus to an amplifying animal which later spread it to more humans. However, empirical evidences gathered to date disprove both theories as none of the samples collected from several animal species from the Wuhan market tested positive for SARS-CoV-2. Contrary to the postulated theories, several of the environmental samples collected from the market tested positive for the virus [72].

This review paper aims to provide an update about SARS-CoV-2 infection at the human-animal interface by reviewing the postulated theories and the collected pieces of evidences ever since the beginning of the pandemic about emergence and transmission of SARS-CoV-2 from animals to human and among human themselves. In addition, the manuscript attempts to comprehend and summarize the current measures undertaken to control and prevent the SARS-CoV-2 infection at the human-animal level and its possible implications on the global public health situation.

### 1.2 Molecular genetic evolution and relatedness of SARS-CoV-2

The first known SARS-CoV-2 isolated from human being shared about 96.2% sequence identity/homology with the closest beta coronaviruses isolated from multiple species of *Rhinolophus* genus bats [73, 74]. Furthermore, the virus shares 96.3% genomic sequence with Bat-CoV-RaTG13 previously detected in the intermediate host horseshoe bat (*Rhinolophus affinis*) in Southwest China's Yunnan Province [75]. SARS-CoV-2 was shown to have about 79.6% similarity with SARS-CoV [74]. Reported for the first time in China in 2019, coronaviruses isolated from Malayan pangolins (*Manis javanica*) are 99% genetically identical to the SARS-CoV-2 [76]. A third study reported that SARS-CoV-2 bears more similarity to Beta CoV/bat/Yunnan/RaTG13/2013 virus, and only 92.4% to the pangolin coronavirus [77]. These findings suggest a zoonotic origin of SARS-CoV-2 [78] and a common ancestral background of these viruses. However, as a member of sarbecovirus subgenus, almost half of the SARS-CoV-2 genome is genetically different from the other subgenus members [79, 80]. The divergences were estimated as 1948 (95% HPD: 1879–1999), 1969 (95% HPD: 1930–2000), and 1982 (95% HPD: 1948–2009) [80] suggesting a new lineage and ridding the possibility of recent recombination [79, 80]. Considering that 90% of the SARS-like viruses originating in bats have been isolated from the *Rhinolophus* genus [77, 81], researchers estimated SARS-CoV-2 divergence history as much as 40–70 years ago [82].

### 1.3 Environmental contamination and stability of SARS-CoV-2

A SARS-CoV-2-infected human could excrete the virus through oral (saliva), respiratory (breath or aerosol) discharge, conjunctiva/ocular (tears) routes, digestive tract (faeces) and via contact with contaminated blood. Vertical transmission among human had also been observed and demonstrated by the expression of S and N proteins in a COVID-19 pregnant woman's placenta, thus confirming SARS-CoV-2 viral RNA positive newborn [83]. Experimentally, the virus was detected in ferrets' urine up to day 8 of infection, however, with low viral loads compared to nasal washings or faecal samples [84].

The ability of the excreted SARS-CoV-2 to remain viable in the environment has also been extensively investigated. In general, the SARS-CoV-2 and other SARS-CoV-2-like viruses appear to be stable and persistent in the environment and fomites thus raising challenges for disinfecting contaminated surfaces. Like-SARS-CoV, some investigations concluded that SARS-CoV-2 could remain on surfaces and aerosol droplets for up to 3 h [85, 86]. On metal or plastic surface, both viruses can remain viable for up to 4 days, however, with a significant reduction in titer. SARS-CoV is thermally inactivated at 60 °C for at least 30 min in protein medium compared to 56 °C in protein-free media [86]. While SARS-CoV-2 remains stable at 0 °C, a study on its environmental stability indicates that it is likely inactivated after 10 min of exposure to 56 °C or more or within less than 5 min at 70 °C [85].

Several chemical decontaminants have been prescribed to efficiently inactive SARS-CoV-2 on surfaces. The virus is rendered inactive by lipid solvents; the most efficient of which being alcoholic compounds including propanol (70%–100% propyl alcohol) or ethanol (70% ethyl alcohol) applied for a minimum of 30 s. Other effective disinfectant chemicals include ether (75%), peroxyacetic acid, chloroform and quaternary ammonium or phenolic compounds with a minimum contact time of 10 min. Chemicals like wine vinegar applied for 1 min, sodium chlorite for 1–2 min and hydrogen peroxide for 2 min have also shown to have disinfecting properties against the SARS-CoV-2. Povidone-iodine 7.5% and chlorhexidine 0.05% [85, 86] are also found to effectively neutralize the virus within 5 min of application.

Available information indicates that SARS-CoV-2 is easy to isolate and culture into Vero cell lines [87, 88, 89, 90], nevertheless, successful inactivation of SARS-CoV-2 should only be confirmed by failure to isolate the virus and should not be judged by viral genome detection.

## 2 Sources of the SARS-CoV-2

Extensive research has been conducted on SARS-CoV-2's possible source of origin and the crossing of species barrier ever since the first identification of the case of COVID-19. Although the SARS-COV-2 virus is strongly postulated to have originated from animals, scientist yet to identify a specific animal species and to conclude whether such animal acts as intermediate host or reservoir of the virus. Nonetheless, the early investigations of the Huanan Seafood Market, Wuhan, China, suspected both seafood and wild animals as the potential sources of the outbreak. Several theories have been put forward to explain the introduction of the origin of the SARS-CoV-2. All these theories move around the role of wild animals sold at the Wuhan market. The most popular theory proposes that the virus was transmitted to the human population from an animal source at the market. The second theory suggests the introduction of the virus to the market from an infected human who transferred the virus to animals where it was amplified before being transferred back to human populations. However, while several of the environmental swab samples tested positive allowing for the virus to be isolated [71], none of the samples taken from several species of animals at the market were found positive for the SARS-CoV-2.

The implication of civet cats as intermediate hosts for SARS-CoV and camels for MERS-CoV [91, 92] has given credence to the fact that animals can serve as a reservoir for SARS-CoV-2. Owing to the genetic similarity especially between SARS-CoV-2 and SARS-CoV, researchers are considering the importance of the intermediate host at the human-animal interface as opposed to focusing solely on the origin of the virus itself. With exception to rats and mice, SARS-CoV-2 likely recognizes ACE-2 orthologues from a diversity of animal species including bats, pigs, ferrets, cats, orangutans and monkeys [93]. The recent investigations tend to attribute the spillover of the virus into human populations to horseshoe bats [81].

### 2.1 The **potential** of SARS-CoV-2 to break through the species barrier and zoo animals

In a zoo, animals are under standard constant health surveillance, good management conditions and minimum stress stimuli, therefor the chances for SARS-CoV-2 to cross the species barrier are highly reduced. As well, unlike the condition in the wildlife markets where there are poor environmental conditions for different animal keeping, slaughtering and meat storage, close contact of multiple species is rare in the wild rendering the spread of SARS-CoV-2 among animals to a minimum. For the virus to cross the species barrier, certain conditions should be fulfilled. These include the presence of an infected animal, infectious secretions and close contact of the animal, possibly repetitively with the sources of infection. Close contact is conducive to stress the infected-animal to shed substantial amount of the virus, which might raise the chances of CoVs to cross the species barrier [94] and hypothetically have a higher opportunity to mutate owing to its unique large and long RNA nucleic material [81]. Interestingly, it was observed that the mutation rates of CoVs are almost similar to the other viral families. Nevertheless, it has been shown recently that some CoVs can regulate some degree of replication under certain environmental circumstances making them more complex adaptors to their settings [95]. CoVs have demonstrated their capacity to potentiality cross the species barriers during the SARS outbreak in 2002, thanks to their dual ability to recombine and spontaneously mutate.

### 2.2 Exposure of wildlife to SARS-CoVs and SARS-CoV-like viruses

During the 2003–2004 outbreak of SARS-CoV, exotic animals were considered the most likely source of infection [96]. SARS-CoV had been known to cross the species barrier by infecting the Himalayan palm civets (*Pagkuma larvata*), raccoon dogs (*Nyctereutes procyonoides*) [97] and Chinese ferret badgers (*Melogale moschata*) and humans [98]. Samples from the Himalayan palm civets (*Paguma larvata*) revealed viruses identical (99.5%) to SARS-CoV genomes whereas serum from Raccoon dogs (*Nyctereutes procyonoides*) showed anti-SARS-CoV neutralizing antibodies [98]. Additionally, SARS-CoV ribonucleic acid (RNA) was detected in *P. larvata* in animal markets and farms [99]. During laboratory investigations, *P. larvata* was successfully infected with the virus and was reported to be equally susceptible to 2 different SARS-CoV therefore providing the previous assumption that civets can play a role in intermediate transmission of SARS-CoV from animals to humans [100]. In China, anti-SARS-CoV antibodies were detected in the lesser horseshoe bat (*Rhinolophus pussilus*), the great-eared horseshoe bat (*R. macrotis*), Pearson's horseshoe bat (*R. pearsoni*), Chinese horseshoe bat (*R. sinicus*) and fruit bat (*Rousettus leschenaultia*). No antibodies were detected in other bat species, including the lesser dog-faced fruit bat (*Cynopterus sphinx*), the mouse-eared bats (*Myotis ricketti*, *Myotis altarium* and *Myotis chinensis*), the noctutule bats (*Nyctalus plancyi* and *N. noctula*), the great round leaf bat (*Hipposideros armiger*) and the intermediate horseshoe bat (*R. affinus*) [101].

### 2.3 Wildlife movement and wild meat consumption

China is one of the largest consumers of wildlife products [102], and the exploitation of wildlife for food and traditional medicines are quite prevalent [103]. This practice has promoted the illegal trade of wild animals including Chinese pangolin (*Manis pentadactyla*) and tiger (*Panthera tigris*), bringing these species to the verge of extinction [104]. Because of that hunters, traders, and consumers are expected come in either direct or indirect contact with wild animals. Further, both wild and domestic animals come in contact with each other in market areas. Studies suggest that more than 1 billion instances of direct and indirect contacts occur among wild and domestic animals and humans from wildlife trade annually [105].

Among animals, pangolins are the world's most trafficked mammal, [106]. The top ten countries and territories most involved in animal trafficking incidents are, China, Vietnam, Malaysia, Hong Kong Special Administrative Region, Thailand, Lao People's Democratic Republic (Lao PDR), Indonesia, US, Nigeria, and Germany. China is identified as the most common destination for international trafficking of live pangolin and its scales [106]. Different species of live pangolins including *M. gigantea*, *M. tetradactyla* and mostly *M. tricuspis* are smuggled from Togo, Nigeria, Congo, the Democratic Republic of the Congo and Uganda to China, Lao PDR and Vietnam for captive-breeding purposes [107]. Pangolin's meat is a popular food and its body parts are essential ingredient for traditional Chines medicine [108, 109, 110]. China is also reported to be the biggest market for illegally smuggled skins, bones, gallbladder, blood and meat of big cats and probably live specimen [111, 112]. Several wet markets also sell cats, raccoons and other wildlife animals [113].

## 3 SARS-CoV-2 host range

Considering the four genera of coronaviruses, bats have been shown to support the evolutionary history and dissemination of Alpha- and Beta-coronaviruses, while birds are carriers of Gamma- and Delta-coronaviruses [114]. Among the four identified lineages of the Beta-coronavirus, human, murine, porcine, equine, rabbit, camel, bovine and antelopes are recognized to support replication of lineage A; human, bats and palm civet support replication of lineage B; camels and bats support replication of lineage C; and bats alone support replication of lineage D [115].

### 3.1 Could SARS-CoV-2 infect animals?

Due to close contact between human and domestic animals, mostly cats and dogs; there is a great risk of spread of SARS-CoV-2 among domesticated animals. Although CoVs are well adapted to cross the inter-species barriers, only a few members of the virus family have managed to do that (SARS-CoV infects human, civet cats, raccoon dogs, horseshoe bat, swine; MERS-CoV infects human, bats, hedgehogs, camels; Bov-CoV infects cattle, wild ruminants, camelids, dogs, occasionally humans).

**Figure 1:**
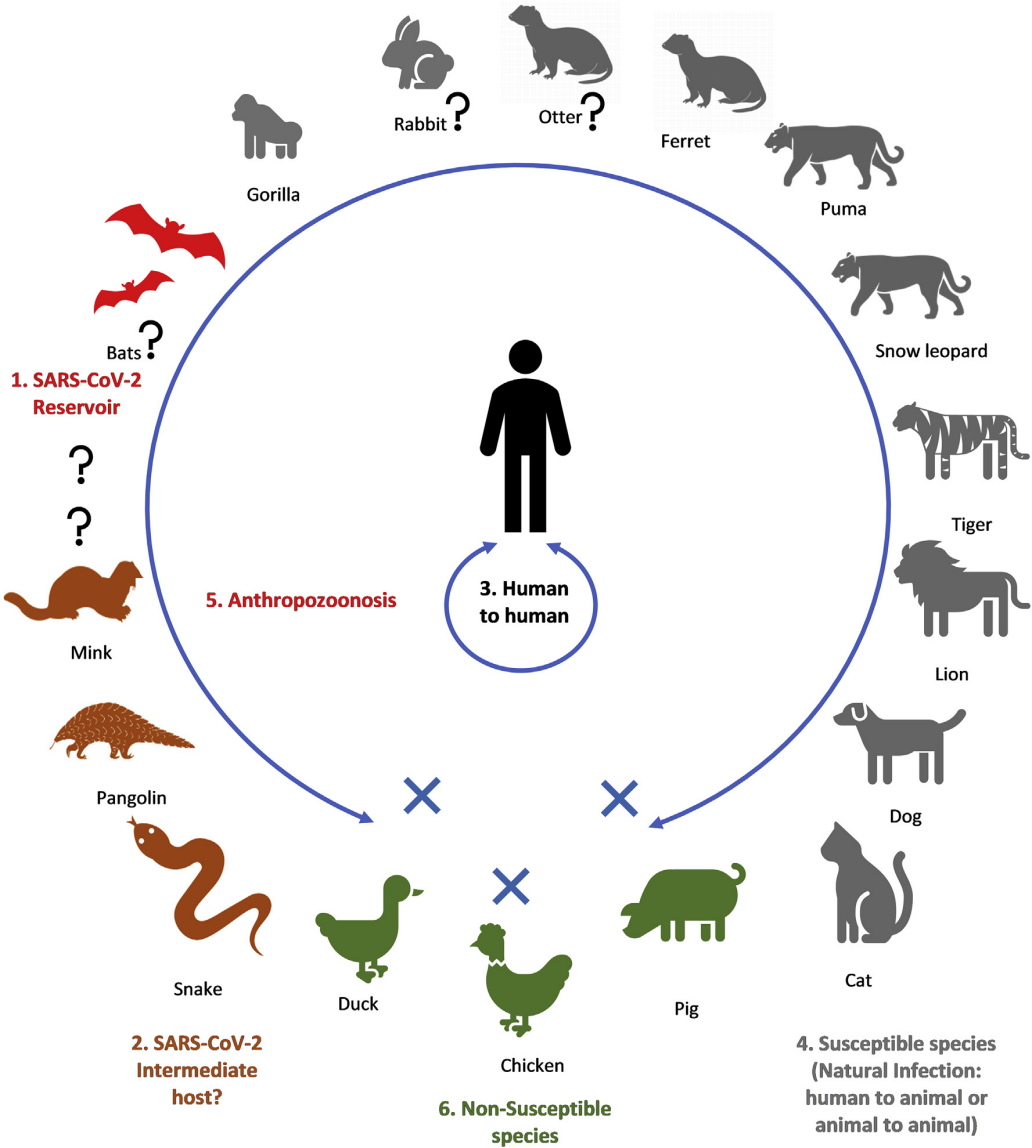
Schematic diagramatic representation showing to date confirmed and specualted SARS-CoV-2 spilling from an animal reservior (1) to inermediate hosts, mostly minks? (2), then to humans (3), then from humans to other susceptible animals (4) or spilling back (anthropozoonosis) to minks (5). Some animals contarcted the disease from other infected animals (rabbits and otters). SARS-CoV-2 non-susceptible animals (6) are also shown.

To date, only eleven animal species are known to become infected with SARS-CoV-2 [Figure 1]. At least 30 countries throughout Europe, South America, North America, Asia and Africa reported the infection [116]. Some of which were experimentally infected, however, little is known about the spread of the virus among free wildlife animals. Moreover, there is an ever-increasing instance of natural exposure of different farm and zoo animals to SARS-CoV-2. Contrary to the common believe, the inter-species infection of the virus does not necessary result in a clinical disease, most COVID-19 human cases result in subclinical or mild infections.

#### 3.1.1 SARS-CoV-2 natural infection in animals

Naturally-acquired infection of a SARS-CoV-2 susceptible animal requires close contact with COVID-19 infected humans. Among domesticated animal settings cats (*Felis catus*) [117, 118], dogs (*Canis lupus familiaris*) [119, 120] and ferrets (*Mustela putorius furo*) [120, 121] were respectively the first reported domestic animals to have naturally acquired SARS-CoV-2 infection. Among these three animals, cats [122] and ferrets [121, 122] were reported highly susceptible to SARS-CoV-2 while dogs got only mild infection [122]. Among captivated zoo animals, lions (*Panthera leo*) [118, 123], tigers (*Panthera tigris*) [123, 124, 125], minks (*Neovison vison*) [126, 127, 128, 129], snow leopard (*Panthera uncia*) [130] puma (*Puma concolor*) [131], gorilla (*Gorilla gorilla*) [132, 133] and otters (*Lutra canadensis*) [134] were also found to be naturally infected.

#### 3.1.2 SARS-CoV-2 experimental infection in animals

Several species of animal were exposed to SARS-CoV-2 under laboratory-controlled environment. Each species showed varying degree of clinical response and virus shedding [135, 136]. These include domestic cats (*Felis catus*), dogs (*Canis lupus familiaris*), ferrets (*Mustela putorius furo*) [121, 122, 137, 138], American mink (*Neovison vison*) [139], Syrian hamsters (*Mesocricetus auratus*) [140, 141], Egyptian fruit bats (*Rousettus aegyptiacus*) [137], Roborovski's dwarf hamster (*Phodopus roborovskii*) [142], deer mice (*Peromyscus maniculatus*) [143, 144], bushy-tailed woodrats (*Neotoma cinerea*) and striped skunks (*Mephitis mephitis*) [145], bank voles (*Myodes glareolus*) [146], rhesus macaques (*Macaca mulatta*) [147, 148, 149], cynomolgus macaques (*M. fascicularis*) [152, 154], African green monkeys (*Chlorocebus* sp.) [155], Chinese tree shrews (*Tupaia belangeri chinensis*) [156], common marmosets (*Callithrix jacchus*) [149], raccoon dogs (*Nyctereutes procyonoides*) [153], raccoons (*Procyon lotor*) [154], white-tailed deer (*Odocoileus virginianus*) [155], laboratory rabbits (*Oryctolagus cuniculus*) [156] and transgenic mice (*Mus musculus*) [157].

However, some species of animals were showed exemplary resistance to experimental infection with SARS-CoV-2. To date, these include cattle (*Bos taurus*) [158], chicken, ducks, and pigs [159, 160], cottontail rabbits (*Sylvilagus* sp.) [95, 145], fox squirrels (*Sciurus niger*), Wyoming ground squirrels (*Urocitellus elegans*), black-tailed prairie dogs (*Cynomys ludovicianus*) [145], house mice (*Mus musculus*) and big brown bats (*Eptesicus fuscus*) [145, 160]. Studies are underway to better understand the susceptibility of animals to SARS-CoV-2 and the infection dynamics among different animal species.

The sensitivity of a given species to get infected by SARS-CoV-2 was assessed *in vitro* using various mammalian cells and computer simulated predictions. The computer prediction models are based on evaluating the potentiality of the host cell receptor domain to bind to the angiotensin converting enzyme2 receptors (RBD/ACE2) [77, 161, 162, 163]. The combination of the virus cultivation and the results of the computer prediction models used in 4 different studies are shown in Table 1. Animal models demonstrating the human ACE2 (hACE2) gene were also used to assess infectivity to SARS-CoV-2 [164, 165].

**Table 1:** Data showing experimental and computer predictary results of some SARS-CoV-2 susceptible animal species.

Extant knowledge about species sensitivity to SARS-CoV-2	Computer prediction of receptor binding Score/5: matched Amino acid		In vivo experimental infection success	
Species	Infected Cell		Viral Particle enter	
Horseshoe Bat [160,1170,198]	YES	YES	Likely	Not Yet
Daubenton's bat [158]	?	NO	?	Not Yet
Vampire bat [158]	?	?	Likely (4/5)	Not Yet
Cynomolgus monkey [[152], [153], [154],^168^]	?	YES	Likely (5/5)	Not Yet
Anubis baboon [166]	?	?	Likely (5/5)	Not Yet
Orangutan [166]	?	?	Likely (5/5)	Not Yet
Chimpanzee [166,167]	?	?	Likely (5/5)	Not Yet
Swine [154,166,169]	YES	NO	Likely (5/5)	Not Yet
Cattle [162,166,168]	?	NO	Likely (4/5)	Not Yet
Elephant [166]	?	?	Unlikely (3/5)	Not Yet
Camel [166]	?	?	?	Not Yet
Mouse [158,161,169]	NO	NO	Unlikely (2/5)	
Rat [166]	?	?	Unlikely (3/5)	
Chinese Hamster [167]	?	NO	Likely (4/5)	
Guinea Pig [169]	?	?	Unlikely (2/5)	
Dog [123,143,[166], [167], [168], [169]]	?	YES	Likely (3/5)	No but seroconverted
Domestic cat [164,167,192]	?	?	Likely (3/5)	Yes + transmission to other cats
Civet cat [164,166]	YES	?	Likely	Not Yet
Meerkat [165]	?	?	Unlikely (2/5)	Not Yet
Raccoon [155]	?		Unlikely (2/5)	Not Yet

### 3.2 Could the SARS-CoV-2 be transmitted from animal to human?

The initially reported cases of SARS-CoV and COVID-19 were observed close to the Chinese New Year, the time when the demand and trade of livestock and wild animals’ peaks to the highest levels. This point in the time is considered a high-risk period for spillover of zoonotic pathogens, especially given the constricted setting of the markets which facilitates air-borne and fomite-borne infections [166]. The model-based analysis for anthropozoonosis did not confirm that either bats or pangolins were the actual source for human SARS-CoV-2. However, it was alarming when it was observed that SARS-CoV-2 infection was discovered in animals; and when it was observed that the virus could induce both intra and interspecies infection, a case reported among cats, ferrets, rabbits, minks [128], Golden Syrian hamsters [140, 141] and cynomolgus and rhesus macaques [151]. The potential spread of the SARS-CoV-2 among susceptible minks [167] and the subsequent identification of SARS-CoV-2 mink related variant [168] indicated rapid adaptation of the virus to animal hosts and suggested that mink could act as an intermediate host for SARS-CoV-2 [169]. These findings were confirmed later observing that the virus could thrive and attain back and forth replication inducing anthroprozoonotic transmission [170]. Fortunately, unlike human-related mutants, the mink mutant proved not to be too dangerous [171]. However, recent studies advised more elaborated investigations about SARS-CoV-2 infections in minks at the human-animal interface [169, 172].

#### 3.2.1 Likelihood of SARS-CoV-2 to infect human from infected domestic animals and birds

Reports from the initial outbreak of COVID-19 suggested that 27 out of 41 cases had history of visiting Huanan seafood market in China [173] where various livestock and wild animals and their carcasses were up for display and sale. The display was performed in a market of more than 1,000 shops within a proximity of 50,000 square meters [174]. However, the China Animal Health and Epidemiology Centre (CAHEC) of the Ministry of Science and Technology, People's Republic of China announced negative results testing more than 4,800 SARS-CoV-2-suspected animal samples collected from pigs, poultry, dogs and cats [175]. While recent reports revealed human to animal SARS-CoV-2 transmission, no data on domestic animal species infected with SARS-CoV-2 or linked to SARS-CoV-2 exposure in humans was available at Wuhan epidemic. Data on exposure of livestock animals viz. ovine, bovine, equine, camelid and swine were insufficient to assess the risk of exposure.

#### 3.2.2 Likelihood of SARS-CoV-2 to infect human from infected wildlife mammals or birds

A variety of live wild animals are normally sold in a local indoors seafood market of Wuhan, China, including, hedgehogs, badgers, snakes, and turtledoves (*Steptoplia turtur*) [175]. None of the animal samples collected from this market tested positive for SARS-CoV-2 even though SARS-COV-2-infected cases working in the vicinity of this market had contacted these animals. With no evidence that this market was the site of the initial zoonotic spillover, some of the early-infected human cases had no even epi-link with this market.

#### 3.2.3 Likelihood of SARS-CoV-2 to infect human from infected wildlife produce

As mentioned before, the unhygienic conditions of whole markets and retail stores create an ideal environment for cross contamination enabling the virus to jump to yet unidentified animal hosts. Contamination of the hands with SARS-CoV-2 contaminated fluids or excretions with the potential for subsequent hand to nose infection of humans is highly expected. Recognizing the distribution of the binding receptors that support invasion of SARS-CoV-2 into a new host, the role of animal carcasses including raw meat and organs to be a source for SARS-CoV-2 infection must also be taken into consideration. While the ACE2 receptors are essential to support SARS-CoV-2 replication [176], though not all of them are supporting infection. However, the consumption of respiratory, digestive and reproductive organs that might express ACE2 receptors should be guarded. Identification of ACE2 SARS-CoV-2 receptors into different animal organs and carcass might help to identify a possible potential animal produce as a source of SARS-CoV-2 infection. To date, no evidence supports milk can be a source for SARS-CoV-2 infection. While no pieces of evidences were shown to prove the Chinese authority hypotheses of the introduction of SARS-CoV-2 infection into China by multiple frozen fish imports, the assumption should be considered.

#### 3.2.4 Likelihood humans to be infected from other SARS-CoV-2-contaminated sources

The fact that SARS-CoV-2 replicates in the respiratory and digestive tract and several other human and animal systems is considered a major environmental contaminant concern. Hospitalized SARS-CoV-2-infected patients could be potential sources of infection contaminating air, medical equipment, daily-used objects and others [177]. Owing to the excretion of the virus into human excreta environmental non-droplet transmission routes should be concerned, and warranted the possibility of faecal-oral community transmission [178]. Due to the excretion of the virus into human excreta, contamination of water, wastewater and sewage sludges with SARS-CoV-2 might occur [179, 180]. SARS-CoV-2 was isolated from untreated wastewater sources in Australia [181] and a commercial travelling aircraft and cruise ship wastewater [182]. In a study conducted in Brazil, SARS-CoV-2 was also detected from 41.6% (5/12) raw sewage treatment plants [183]. In a different study performed in Spain, Chavarria-Miró et al 2021 [184] detected the SARS-CoV-2 RNA in a sample collected 41 days before the declaration of the first COVID-19 case. Further, their modulated investigation on the total number of shedders estimated 2.0–6.5% prevalence rate among asymptomatic infected individuals. An up dated summary of the investigations showing concerns in SARS-CoV-2-contaminated water sources was given by the European Food Safety Authority and European Centre for Disease Prevention and Control report of 2021 [135]. Several studies advocated waste-based epidemiology as a valuable early warning tool to track SARS-CoV-2 circulation [180, 184] and a measure to evaluate public health safety in a community [185, 186]. However, as an alternative, a survey of rivers was suggested to evaluate the contamination capacity with SARS-CoV-2 in poor community settings [187]. The impact and consequences of this contamination were found to be significant as described later by Patel et al 2021 [188].

## 4 Knowledge gap

To advise an effective counter measures against SARS-CoV-2, it is vital to develop a precise risk assessment framework. Towards this end, there are some crucial gaps in the epidemiological maps of the disease which needs our attention. Some of these gaps are: 1. Application of a one-health assessment to tackle COVID-19 human cases based on: i. Investigating each COVID-19 human case for a history of contact with animals. For this, we recommend employing methods with a high degree of certainty such as ex-vivo tissue respiratory explants. ii. Undertaking high-end molecular studies to enable further virus characterization and to link the suspect primary human cases to the infected animals iii. Collecting data on factors that may increase the risk of animal-exposed human cases and vice versa iv. Conducting field studies to test exposed communities and occupations across the human-animal-environment interface using precise serological methods. 2. Conduction of field studies to investigate potential and intermediate hosts and understand the relationships between different host populations that may facilitate SARS-CoV-2 spillover. This is expected to be carried through: i Targeting domestic and wild animal species that were reportedly sold at the Huanan Seafood Market in Wuhan City and also those that have spike protein sequence data compatible with SARS-CoV-2 receptors. Additional animal species that have had a potential involvement in SARS-CoV-2 transmission and compatible ACE2 receptors could also be targeted. ii. Focusing on animal populations in China and South East Asia, particularly in countries with extensive formal and informal animal trade with China. iii. Follow up of the serologically positive animal species with molecular testing, such as pan-coronavirus family PCR and specific SARS-CoV-2 PCR assays for positive cases. vi. Identifying the transmission pathways between animals, humans, and the environment. 3. Conduction of studies to investigate SARS-CoV-2 survival in the environment, including after undertaking sanitization activities. 4. Study of human behavior that may lead to increased exposure risk to SARS-CoV-2.

## 5 Preventing and controlling SARS-CoV-2 infection at the human-animal interface

It is essential to prevent or curb the transmission of SARS-CoV-2 between humans and susceptible animals before it becomes a major concern. This could be achieved by implementing the national risk reduction strategies. Based on the most current available epidemiological information about COVID-19 and SARS-CoV-2, social distancing, masking, vaccination, and personal hygiene measures are the best means to prevent the spread of infection among human. While the OIE was seeing insignificance on the role of pets on SARS-CoV-2 infection [189], the spread of SARS-CoV-2 among several animals and the nature of the infection among some warrant attention. The recent outbreaks among minks [167, 168, 170, 172] and the reporting of anthropozoonosis [172] with development of mink variant [168] should receive further attention [169].

On the other hand, the continuously evolving nature of SARS-CoV-2 and the news of newer variants now and then warrants consideration to continuously undertake controlling measures of proven efficacy. While it might have social and economic impacts, blanket reduction of contact with animals is recommended. Continuous conduction of sero-surveillance among animals is the first exercise to be done to investigate the capability of SARS-CoV-2 to cross the species barriers and to delineate the complete epidemiology of the disease. Further, undertaking continuous sero-surveillance will enable agencies to understand the capacity of the virus to replicate in different animal tissues. Conduction of retrospective sero-investigations is important to trace back the exposure history and entity. The use of molecular tools to screen SARS-CoV-2 seropositive cases, clinically suspected animals, and SARS-CoV-2 fossil animal samples are effective approaches to rapidly detect SARS-CoV-2 samples. Using of the high throughput next generation sequencing technology of the whole genome of the virus from the positive RT-PCR samples is the best approach to cast light on the molecular epidemiology of the virus and to differentiate the intermediate host animals from SARS-CoV-2 dead-end hosts. Provision of sufficient bioinformatics and analysis for the molecularly positive and sequenced samples is important to uncover the direction of each infection. Quarantining of the infected animals and evacuation of SARS-CoV-2-infected animal closures or farms are important. The adoption of culling as a policy tool is effective strategy to cut down virus circulation. Nonetheless, a culling policy will only work if there is a policy to compensate the owners and farmers of the infected animals to contribute to the control programs [190]. Under certain conditions, a partial or complete ban on the animal movement and trading might also be effective. The establishment of a network communication system is essential to exchange data, forecast information and share experiences. Moreover, the development of education programs to raise awareness of the community on the issue is an essential step toward successful mitigation of COVID-19 in the long-term.

Since vaccination is the most cost-effective measure available for the control of the disease, there are huge efforts to produce newer vaccines against the COVID-19. Only a handful of totals under developed vaccines are approved to date, however, none of them has the collective desired characteristics to protect from infection with SARS-CoV-2. All available vaccines provide effective protective against infection with the alpha-, beta- and gamma strains, however, their efficacy against the latest and most virulent delta strain is uncertain.

The reported outbreaks among different individual animal species contracting SARS-CoV-2 infection from humans [117, 118, 120, 123, 124, 125, 126, 128, 130, 131, 132, 133] and their potential to sustain intra- and inter species circulation of the virus [117, 121, 128, 138, 167] establishing anthropozoonosis [170] calls for more practical and cost-effective means to control and prevent SARS-CoV-2 spread among animal populations rather than culling policy. However, the recently documented ability of the virus to mutate into new variants coupled with efforts to fully understand the replicative behavior of SARS-CoV-2 in different animal species would provide a future challenge for developing an effective vaccine for the animal population.

## 6 Conclusion and future prospects

To date, SARS-CoV-2 is considered to have emerged from a bat coronavirus reservoir. Despite the extensive global efforts to combat COVID-19, scientist have not yet been able to delineate the epidemiology of the disease. This is proving to be problematic in devising effective preventive and controlling measures. The continuous evolution of the original strain of the SARS-CoV-2 into a variety of highly infectious mutants and its ability to infect a variety of domestic and wild animals reflects the ability of SARS-CoV-2 to rapidly adapt to and survive different environmental conditions and to replicate into different human and animal organ systems. Many animals, especially wildlife carnivorous, were identified to have ACE2 receptors and were classified as at-risk to SARS-CoV-2 infection and could potentially act as intermediate hosts and/or to establish anthroprozoonosis. In this respect, the role of most human in-contact animals, especially the companion animals should be guarded. Further investigations considering the host body temperature, level of receptor expression, existence of co-receptor, restriction factors, and genetic background [191, 192] are necessary to identify which animal act as naturally primary and/or intermediate host. Further studies to identify SARS-CoV-2 at-risk animals would help recognition of animal models required for evaluating the future vaccines and the anti-SARS-CoV-2 remedies.

Since the real natural cycle of this virus needs elucidation, further studies at the host-virus interaction levels are necessary to predict the virus response to the host's immune system. Such studies could cast light on the ability of the virus to re-infect vaccine-induced and naturally immune individuals. For animals, there are two policy options to prevent the spread of SARS-COV-2 infection; vaccination of the susceptible animals and culling of the infected. A national strategy should be formulated to evaluate the advantages and disadvantages of both options. Holistic approaches to investigate the genetic, evolutionary and epidemiological nature of SARS-CoV-2 to bridge the public health gaps created by the infection are immediately required [193]. After reviewing the existing literature on the genetic, biology and epidemiology of SARS-CoV-2, it seems that the “One Health” approach is the most efficient framework to appropriately address the colossal challenge posed by the on-going SARS-CoV-2 pandemic.

## Declarations

### Author contribution statement

All authors listed have significantly contributed to the development and the writing of this article.

### Funding statement

This research did not receive any specific grant from funding agencies in the public, commercial, or not-for-profit sectors.

### Data availability statement

Data included in article/supplementary material/referenced in article.

### Declaration of interests statement

The authors declare no conflict of interest.

### Additional information

No additional information is available for this paper.
